# Supplementary material for: Epidemiology of atrial fibrillation in the All of Us Research Program
Source: PLoS One. 2022 Mar 16;17(3):e0265498. doi: 10.1371/journal.pone.0265498 (PMC8926244; doi:10.1371/journal.pone.0265498)
Supplement: S1 Table — (DOCX) [file pone.0265498.s001.docx]

Supplementary Table I. Concept IDs used to identify atrial fibrillation in the electronic health records, *All of Us* Research Program, 2017-2019

| **Description** | **OMOP Concept ID** | **SNOMED Code** |
| --- | --- | --- |
| Atrial fibrillation | 313217 | 49436004 |
| Atrial fibrillation and flutter | 4108832 | 195080001 |
| Atrial fibrillation with rapid ventricular response | 44782442 | 120041000119109 |
| Chronic atrial fibrillation | 4141360 | 426749004 |
| Controlled atrial fibrillation | 4117112 | 300996004 |
| ECG: atrial fibrillation | 4064452 | 164889003 |
| Lone atrial fibrillation | 4119601 | 233910005 |
| Paroxysmal atrial fibrillation | 4154290 | 282825002 |
| Permanent atrial fibrillation | 4232691 | 440028005 |
| Persistent atrial fibrillation | 4232697 | 440059007 |
| Rapid atrial fibrillation | 4199501 | 314208002 |

OMOP: Observational Medical Outcomes Partnership; SNOMED: Systematized Nomenclature of Medicine.
