# Supplementary material for: Epidemiology of atrial fibrillation in the All of Us Research Program
Source: PLoS One. 2022 Mar 16;17(3):e0265498. doi: 10.1371/journal.pone.0265498 (PMC8926244; doi:10.1371/journal.pone.0265498)
Supplement: S2 Table — Values correspond to mean (standard deviation) or N (%), All of Us Research Program 2017–2019. (DOCX) [file pone.0265498.s002.docx]

Supplementary Table II. Participant characteristics by study component participation. Values correspond to mean (standard deviation) or N (%), *All of Us* Research Program 2017-2019

|  | **Overall** | **Both EHR and survey data** | **Only EHR data** | **Only survey data** | **No EHR, no survey data** |
| --- | --- | --- | --- | --- | --- |
| N | 173,101 | 20,683 | 71,636 | 14,800 | 65,982 |
| Age, years | 52 (17) | 55 (16) | 52 (17) | 53 (17) | 51 (17) |
| Female sex | 105,524 (61%) | 13,939 (67%) | 43,500 (61%) | 9,387 (63%) | 38,698 (59%) |
| Race/ethnicity |  |  |  |  |  |
| Non-Hispanic White | 116,241 (67%) | 18,024 (87%) | 43,920 (61%) | 13,182 (89%) | 41,115 (62%) |
| Non-Hispanic Black | 44,993 (26%) | 1,559 (8%) | 22,876 (32%) | 680 (5%) | 19,878 (30%) |
| Non-Hispanic Asian | 7,338 (4%) | 696 (3%) | 2,748 (4%) | 599 (4%) | 3,295 (5%) |
| Hispanic | 4,529 (3%) | 404 (2%) | 2,092 (3%) | 339 (2%) | 1,694 (3%) |
| BMI, kg/m^2^ | 29.0 (6.7) | 28.3 (6.3) | 29.4 (6.8) | 27.9 (6.1) | 29.0 (6.7) |
| SBP, mmHg | 128 (19) | 127 (17) | 128 (19) | 127 (17) | 129 (19) |
| DBP, mmHg | 78 (12) | 77 (11) | 79 (12) | 77 (11) | 79 (12) |
| Ever smoker | 70,320 (41%) | 7,098 (34%) | 32,621 (46%) | 4,690 (32%) | 25,911 (39%) |
| Diabetes | 15,587 (9%) | 2,527 (12%) | 13,060 (18%) | -- | -- |
| Heart failure | 5,177 (3%) | 721 (3%) | 4,456 (6%) | -- | -- |
| CHD | 8,912 (5%) | 1,584 (8%) | 7,328 (10%) | -- | -- |
| Stroke | 490 (0.3%) | 81 (0.4%) | 409 (0.6%) | -- | -- |

BMI: body mass index; CHD: coronary heart disease; DBP: diastolic blood pressure; SBP: systolic blood pressure.
