# Supplementary material for: Epidemiology of atrial fibrillation in the All of Us Research Program
Source: PLoS One. 2022 Mar 16;17(3):e0265498. doi: 10.1371/journal.pone.0265498 (PMC8926244; doi:10.1371/journal.pone.0265498)
Supplement: S3 Table — (DOCX) [file pone.0265498.s003.docx]

Supplementary Table III. Association of age, sex, race/ethnicity and cardiovascular risk factors with prevalent atrial fibrillation among *All of Us* participants with EHR and medical history survey data (N = 20,683), *All of Us* Research Program, 2017-2019

|  | **Odds ratio (95% CI)** |
| --- | --- |
|  | **Model 1*** |
| Age group |  |
| <40 | 1 (Ref.) |
| 40-49 | 2 (1.4, 3.0) |
| 50-59 | 3.4 (2.5, 4.7) |
| 60-69 | 7.7 (5.8, 10.2) |
| 70-79 | 11.9 (8.9, 15.9) |
| 80+ | 24.1 (17.4, 33.5) |
|  |  |
| Sex |  |
| Female | 1 (Ref.) |
| Male | 1.8 (1.6, 2.0) |
|  |  |
| Race / ethnicity |  |
| Hispanic | 0.91 (0.51, 1.6) |
| NH Asian | 0.55 (0.34, 0.89) |
| NH Black | 0.62 (0.46, 0.82) |
| NH White | 1 (Ref.) |
|  |  |
|  | **Model 2**** |
| BMI, per 5 kg/m^2^ | 1.1 (1.1, 1.2) |
| SBP, per 20 mmHg | 0.88 (0.80, 0.96) |
| DBP, per 10 mmHg | 1.1 (1.0, 1.1) |
| Ever smoking | 1.1 (0.97, 1.2) |
| Diabetes | 0.94 (0.80, 1.1) |
| Heart failure | 5.1 (4.2, 6.2) |
| Coronary heart disease | 2.2 (1.9, 2.6) |
| Stroke | 2.6 (1.4, 4.8) |

* Logistic regression including age (categories), sex, and race/ethnicity. ** Logistic regression including age (continuous), sex, race/ethnicity and all other covariates in the table.
