# Supplementary material for: Epidemiology of atrial fibrillation in the All of Us Research Program
Source: PLoS One. 2022 Mar 16;17(3):e0265498. doi: 10.1371/journal.pone.0265498 (PMC8926244; doi:10.1371/journal.pone.0265498)
Supplement: S4 Table — Values correspond to prevalence per 100 persons. (DOCX) [file pone.0265498.s004.docx]

Supplementary Table IV. Age, sex, and race/ethnicity prevalence of AF in the *All of Us* Research Program and selected epidemiologic studies in the United States. Values correspond to prevalence per 100 persons.

| ***All of Us* Research Program** | | | **ATRIA^6^** | | | **Medicare 2007^8^** | |
| --- | --- | --- | --- | --- | --- | --- | --- |
|  | **Survey** | **EHR** |  | **Females** | **Males** |  | |
| **Age** |  |  | **Age** |  |  | **Age** |  |
| <40 | 0.9 | 0.3 | <55 | 0.1 | 0.2 |  |  |
| 40-49 | 2.2 | 1.3 | 55-59 | 0.4 | 0.9 |  |  |
| 50-59 | 3.7 | 2.4 | 60-64 | 1.0 | 1.7 |  |  |
| 60-69 | 7.5 | 5.5 | 65-69 | 1.7 | 3.0 | 66-69 | 3.1 |
| 70-79 | 13.0 | 11.1 | 70-74 | 3.4 | 5.0 | 70-74 | 5.8 |
| 80-89 | 22.0 | 19.1 | 75-79 | 5.0 | 7.3 | 75-79 | 9.4 |
|  |  |  | 80-84 | 7.2 | 10.3 | 80-84 | 13.1 |
|  |  |  | ≥85 | 9.1 | 11.1 | 85-89 | 16.3 |
|  |  |  |  |  |  | ≥90 | 17.5 |
|  |  |  |  |  |  |  |  |
| Sex |  |  | Sex |  |  | Sex |  |
| Female | 4.3 | 3.0 | Female | 1.1 | | Female | 7.4 |
| Male | 8.8 | 6.1 | Male | 0.8 | | Male | 10.4 |
|  |  |  |  |  | |  |  |
| Race/  ethnicity |  |  | Race/  ethnicity |  |  | Race/  ethnicity |  |
| Hispanic | <2.7 | 1.8 |  |  |  |  |  |
| NH Asian | 2.1 | 1.8 |  |  |  |  |  |
| NH Black | 2.8 | 2.2 | Black | 1.5 | | Black | 4.6 |
| NH White | 6.3 | 5.2 | White | 2.2 | | White | 9.1 |

ATRIA: AnTicoagulation and Risk Factors In Atrial Fibrillation. NH: Non-Hispanic
