# Supplementary material for: Epidemiology of atrial fibrillation in the All of Us Research Program
Source: PLoS One. 2022 Mar 16;17(3):e0265498. doi: 10.1371/journal.pone.0265498 (PMC8926244; doi:10.1371/journal.pone.0265498)
Supplement: S5 Table — Values correspond to incidence rate per 1000 person-years. (DOCX) [file pone.0265498.s005.docx]

Supplementary Table V. Age, sex, and race/ethnicity incidence rates of AF in the *All of Us* Research Program and selected epidemiologic studies in the United States. Values correspond to incidence rate per 1000 person-years.

| ***All of Us***  **Research Program** | | **CHS^5^** | | | **FHS^3^** | | | **ARIC study^4^** | | | **Medicare 2007^8^** | |
| --- | --- | --- | --- | --- | --- | --- | --- | --- | --- | --- | --- | --- |
|  |  |  | **Females** | **Males** |  | **Females** | **Males** |  | | |  | |
| **Age** |  | **Age** |  |  | **Age** |  |  | **Age** |  | **Age** | |  |
| <40 | <0.6 |  |  |  |  |  |  |  |  |  | |  |
| 40-49 | <1.2 |  |  |  |  |  |  | 45-49 | 0.6 |  | |  |
| 50-59 | 2.2 |  |  |  | 55-64 | 5 | 7 | 50-59 | 1.8 |  | |  |
| 60-69 | 4.1 | 65-69 | 10.9 | 12.3 | 65-74 | 10 | 18 | 60-69 | 5.3 | 66-69 | | 12.9 |
| 70-79 | 7.6 | 70-74 | 9.1 | 22.8 | 75-84 | 30 | 35 | 70-89 | 12.2 | 70-74 | | 18.8 |
| 80-89 | 12.3 | 75-79 | 23.1 | 34.8 | 85-94 | 65 | 77 | ≥80 | 38.7 | 75-79 | | 28.8 |
|  |  | ≥80 | 25.1 | 58.7 |  |  |  |  |  | 80-84 | | 38.3 |
|  |  |  |  |  |  |  |  |  |  | 85-89 | | 53.5 |
|  |  |  |  |  |  |  |  |  |  | ≥90 | | 68.9 |
|  |  |  |  |  |  |  |  |  |  |  | |  |
| Sex |  | Sex |  |  | Sex |  |  | Sex |  | Sex | |  |
| Female | 2.2 | Female | 14.1 | | Female | 11.3 | | Female | 3.7 | | Female | 24.7 |
| Male | 4.1 | Male | 26.4 | | Male | 14.5 | | Male | 6.1 | | Male | 33.9 |
|  |  |  |  | |  |  | |  |  | |  |  |
| Race/  ethnicity |  | Race/  ethnicity |  |  |  |  |  | Race/  ethnicity |  | Race/  ethnicity | |  |
| Hispanic | <5.9 |  |  |  |  |  |  |  |  |  | |  |
| NH Asian | <4.1 |  |  |  |  |  |  |  |  |  | |  |
| NH Black | 1.5 | Black | 12.0 | |  |  |  | Black | 3.3 | Black | | 22.1 |
| NH White | 3.6 | Non-Black | 19.5 | |  |  |  | White | 5.2 | White | | 29.4 |

ARIC: Atherosclerosis Risk in Communities. CHS: Cardiovascular Health Study. FHS: Framingham Heart Study. NH: Non-Hispanic.
