# Supplementary material for: Epidemiology of atrial fibrillation in the All of Us Research Program
Source: PLoS One. 2022 Mar 16;17(3):e0265498. doi: 10.1371/journal.pone.0265498 (PMC8926244; doi:10.1371/journal.pone.0265498)
Supplement: Attachment — Submitted filename: response to reviewers.docx [file pone.0265498.s006.docx]

**Response to reviewers**

We thank the reviewers for their evaluation of our manuscript. Below we provide a point-by-point response to their comments.

**Reviewer #1**

**“This study proposes to establish the prevalence, incidence, and risk factors for atrial fibrillation in a large cohort. Among 92,318 participants with available EHR data, 3,885 (4.2%) had AF at the time of study enrolment. During a median follow-up of 16 months, 354 new cases of AF were identified among 88,433 eligible participants. This study confirms information already known, but remains interesting. Its main problem concerns recruitment bias and the reliability of the information collected. Indeed, AF was not noted on the physical measurements recorded at enrollment. This would have avoided some false positives and false negatives. Indeed, the way AF is diagnosed, either at enrolment or in the follow-up, inevitably leads to diagnostic errors.”**

We agree with the reviewer that the method of AF ascertainment is imperfect and will likely lead to false positives and negatives. This limitation is highlighted in the discussion.

**“Major concern
1/ You need to be clearer about the number of patients evaluated, throughout the paper.
For example, 35,483 (21%) had completed the medical history survey and 92,318 (53%) had EHR data available at the time of dataset creation. There were 20,683 participants who had both medical history survey and EHR data. So your data is from how many unique patients actually?** **107,118 ?”**

As suggested by the reviewer, we clarify in the text that the analysis eventually excluded those who did not complete the medical history survey or had available EHR data at the time of cohort creation, leaving 107,118 unique participants eventually included in the analyses. This has been corrected in the manuscript throughout.

**“2/ Can you detail the number of 50,822 excluded patients for not meeting inclusion criteria ? This seems like a high number when you start with 223,921 patients. How many because of age? gender? race?”**

Most of the exclusions were due to race not being in the categories Asian, Black/African American, or White, reporting more than one race, or the question not being answered by the participant (n = 49,508.) Exclusions due to other criteria (gender, ethnicity) were less frequent (n = 4,684 and n = 5,661, respectively). Figure 1 has been updated to include these numbers.

**“3/ The result on blood pressure is very surprising. It needs to be better explained in the discussion.”**

We agree with the reviewer about the unexpected nature of the findings. Notably, the inverse association between systolic blood pressure and AF was only seen in the analysis of prevalent AF but not for incident AF. Reverse causation may explain this association, with more strict blood pressure control in individuals who already have a cardiovascular disease including AF. We have added a sentence in the discussion providing this explanation.

**“Minor concerns
1/ Figure 1 should be reworked according to the usual presentation of a flow chart.”**

We have updated Figure 1 with exclusion numbers and improved the formatting.

**“2/ I don't understand why you give the total population statistics in table 1, when you have usable data for only a portion of them in the rest of the study.”**

As suggested by the reviewer we have updated Table 1 providing baseline information on the 107,118 participants who were eventually included in the analysis.

**Reviewer #2**

**“The manuscript aimed to investigate the prevalence, incidence and risk factors of atrial fibrillation in a large, geographically and ethnically diverse cohort in the United States.
The idea is interesting with few elements of novelty.”**

We appreciate the reviewer’s assessment of our manuscript. As stated in the introduction, the goal of this analysis is to determine the validity of *All of Us* Research Program data by replicating associations previously described in other epidemiologic studies, and not to report novel associations. The novelty resides not on the actual findings, but on demonstrating that *All of Us* can be of use to conduct epidemiologic studies of atrial fibrillation.

**“The sample size is adequate but the study suffers from a lack of iconography and the statistical analysis is poor.”**

We think that the statistical approach used is relevant to address the study aims. We would appreciate more details about aspects that the reviewer believes should be improved.

**“The discussion and the reference section needs to be improved. Please cite and discuss the role of comorbidities in atrial fibrillation.”**

As suggested by the reviewer, we provide a more extensive discussion about the role of comorbidities in atrial fibrillation and have added some relevant references.
